# The Role of Cytoskeletal Proteins in the Formation of a Functional In Vitro Blood-Brain Barrier Model

**DOI:** 10.3390/ijms23020742

**Published:** 2022-01-11

**Authors:** Shireen Mentor, Khayelihle Brian Makhathini, David Fisher

**Affiliations:** 1 Neurobiology Research Group, Department of Medical Biosciences, University of the Western Cape, Cape Town 7535, South Africa; 2746944@myuwc.ac.za (S.M.); kmakhathini@uwc.ac.za (K.B.M.); 2 School of Health Professions, University of Missouri, Columbia, MO 65211, USA

**Keywords:** nanotubules, Cytochalasin D, Nocodazole, brain endothelial cells, barrier-genesis

## Abstract

The brain capillary endothelium is highly regulatory, maintaining the chemical stability of the brain’s microenvironment. The role of cytoskeletal proteins in tethering nanotubules (TENTs) during barrier-genesis was investigated using the established immortalized mouse brain endothelial cell line (bEnd5) as an *in vitro* blood-brain barrier (BBB) model. The morphology of bEnd5 cells was evaluated using both high-resolution scanning electron microscopy and immunofluorescence to evaluate treatment with depolymerizing agents Cytochalasin D for F-actin filaments and Nocodazole for α-tubulin microtubules. The effects of the depolymerizing agents were investigated on bEnd5 monolayer permeability by measuring the transendothelial electrical resistance (TEER). The data endorsed that during barrier-genesis, F-actin and α-tubulin play a cytoarchitectural role in providing both cell shape dynamics and cytoskeletal structure to TENTs forming across the paracellular space to provide cell-cell engagement. Western blot analysis of the treatments suggested a reduced expression of both proteins, coinciding with a reduction in the rates of cellular proliferation and decreased TEER. The findings endorsed that TENTs provide alignment of the paracellular (PC) spaces and tight junction (TJ) zones to occlude bEnd5 PC spaces. The identification of specific cytoskeletal structures in TENTs endorsed the postulate of their indispensable role in barrier-genesis and the maintenance of regulatory permeability across the BBB.

## 1. Introduction

The brain microvascular endothelial cells (BECs) play a critical role as an interdependent network constituting the basic angioarchitecture of the blood-brain barrier (BBB) [1]. The functionality of the BBB is closely related to the regulation of permeability and entails paracellular (PC) sealing by intercellular tight junction (TJ) protein complexes as a central feature of the barrier’s physical function. How the zones of the TJs are aligned is crucial to their action. Compared to systemic endothelial cells, the apico-lateral expression of the TJs is specific, unique, and depends on the correct orientation of the BECs into the apical and basal domains [1]. The mechanics that are required for two juxtaposed BEC membranes to align in a manner that enables TJ interaction with its counterparts on adjacent cells have recently been described from an ultrastructural perspective, highlighting for the first time, the importance of nanotubules (NTs) in the physical alignment of adjacent BECs by utilizing high-resolution electron microscopy (HREM) [2]. Two types of novel NTs were discovered as a form of direct BEC–cell interaction at the apico-lateral domains of BECs and can be categorically differentiated into (i) nanovesicle (NV)-induced tunneling NTs (TUNTs) and (ii) tethering NTs (TENTs). The study illuminates the ultrastructural prowess of these NTs to generate an extracellular PC scaffolding, which facilitates TJ interaction and PC occlusion. Moreover, TENTs were described as novel nanostructural cross-bridges that emerged transiently as a supplementary means of direct cell-cell communication during immortalized mouse BEC (bEnd3/bEnd5) monolayer development *in vitro*, which has become a significant feature in redefining the mechanobiology that is involved in TJ localization during *in vitro* BBB establishment.

Although intercellular communication has revolutionized our understanding of barrier-genesis concerning BBB establishment, however, little is known about its nanoscopic development, thus limiting our understanding of the mechanobiology that is associated with its physical functionality.

Defining the BEC ultrastructural interaction is necessary when elucidating: (i) BEC proliferation dynamics, (ii) angiogenesis, and (iii) BEC permeability, which are critical parameters for ensuring a well-regulated barrier interface. It is known that the BBB is established by TJ protein-protein interactions between adjacent BECs [3,4,5,6]. Upon investigating the morphological profile of the BECs on a nanoscale, it was revealed that the barrier interface possesses a complex interactive PC engagement between the adjacent *in vitro* BECs growing in close proximity [2].

BECs are specifically designed to align with each other, enabling zones of apico-lateral, juxtaposed TJs to form a highly restrictive PC shunt to prevent PC permeability across brain capillaries. Furthermore, the regulatory functions of the BECs would be nullified by a permeable PC space [6]. 

In recent years, a novel type of cell-cell communication was observed that was based on the formation of NTs between cells. NT ultrastructures were first reported in pheochromocytoma cells and were described as actin-dependent tubules [7]. Similarly, Ma and Zhou et al. [8] under low-resolution reported the formation of membrane protrusion, which they categorized as primary “cilia”. They were further described as microtubule, tubulin-based structures, protruding from *in vitro* BEC cell surfaces [8].

NT cross-bridge formation is a highly prevalent feature between adjacent BECs during *in vitro* BEC monolayer development [2]. In earlier studies, direct cell-cell communicatory NTs have been described as actin-rich NTs and/or cross-bridge NTs [9]. Although NTs were initially described in rat PC12 cells [7], it has never before been reported to play a functional role in the establishment of the BBB [2]. 

These recent morphological findings prompted the redefinition of the existing comprehension of barrier-genesis. Interacting BECs that line the brain capillaries, accomplish specific barrier roles, namely: regulating transcellular and PC permeability, which affects the transcellular exchange of metabolites and nutrients. This research paper reports on the cytoskeletal molecular structures, which potentially underpin the formation of TENT structure and function enabling it to generate a functional *in vitro* BBB model.

Cytoskeletal proteins such as actin-microfilaments have been closely associated with cellular processes such as migration, endocytosis, cytokinesis, and cytoplasmic projections and thus, it is a critical modulator of cell physiology. Moreover, actin has been reported to be the backbone of such cytoplasmic protrusions [9]. Moreover, Gurke et al. [7] reported that cytoplasmic protrusions evolve into NT-like cross-bridges, which are F-actin dependent [7]. A study by Phng et al. [10] supports these findings, reporting on the role of actin polymerization and its ability to ensure the protrusion of filopodial migration between two ECs in dorsal longitudinal anastomotic vessels and blood vein plexus formation [10]. 

Furthermore, a recent study on microtubules has sparked interest as these structures have been described as surface membrane protrusions on the EC and have been postulated to be critical for the functional role in vascular barrier regulation [8]. In this regard, the literature reports that the destruction of fibroblastic microtubules has a negative impact on protruding actin-rich lamellipodial-based migratory processes. [11,12]. It is further hypothesized that the microtubules are concomitant with the polymerization of actin at the cell leading edges, which induces cytoplasmic projections [11,13].

Thus, TENTs that are generated by BEC surface are membrane NT protrusions that appeared to be integral for the juxtapositioning of BECs to ensure the appropriate intercellular TJ alignment and subsequent occlusion of BEC PC spaces during the establishment of a confluent monolayer. The molecular structures, which underpin TENT cytoskeletal morphology are, however, yet to be investigated. To date, how these TENTs project themselves across the PC space has not yet to be elucidated. This study focused on the functional role of the cytoskeletal architecture of the BEC as a critical feature governing its mechanobiology, and further postulates that the cytoskeletal proteins F-actin and α-tubulin are implicated in molecular underpinning the mechanisms of TENT formation in the bEnd5 cells.

## 2. Results

**Figure 1:**
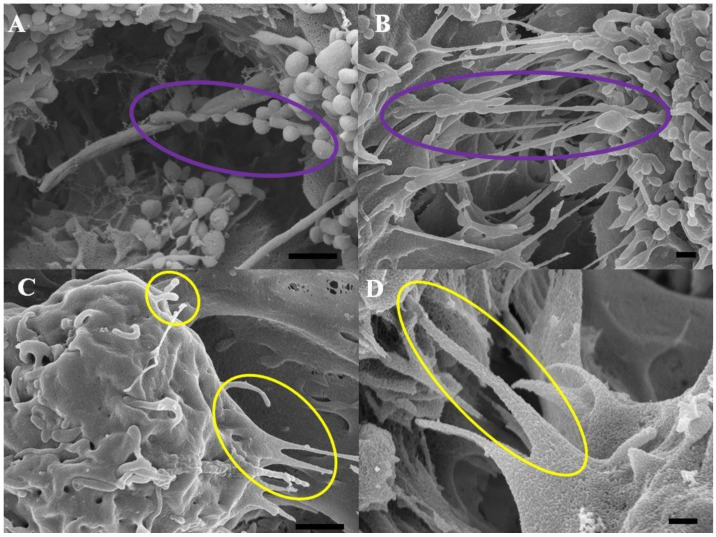
HR-SEM micrographs representing BEC bEnd5 NTs that are subdivided into two types: (**A**) NV-induced TUNTs indicated by the **purple** circles, scale bar 1000 nm; (**B**) TUNT formation and extension across the PC space between adjacent BECs, upon the fusion of multiple NVs, scale bar = 300 nm; (**C**) TENTs formed by the BEC membrane leading edges as indicated by the **yellow** circles, scale bar = 1000 nm; (**D**) A magnified TENT extension across the PC space between two adjacent BECs forming an occluded tent-like covering, scale bar = 300 nm.

The study investigated the structural organization of the BECs, the cytoskeletal molecular underpinnings of microfilaments and microtubules, and their functional role in establishing BEC monolayer integrity via TENT connections. Recent high-resolution scanning electron microscopy (HR-SEM) observations in our laboratory showed that the nanoscopic morphology of the BEC confluent monolayers exhibits cytoplasmic projections, which are continuous with the leading edges of the BEC membrane (See Figure 1 below).

### 2.1. Morphology of BEC Nanotubules at High Resolution

Figure 1A,B, shows the first category of NTs (i.e., TUNTs) which develop during the BEC monolayer establishment. TUNTs were first described in our laboratory and are formed by the fusion of a series of NVs (Figure 1A) into long, continuous cross-bridge TUNTs between adjacent BECs (Figure 1B). Figure 1C, D, shows the second category of NTs which form tether-like NT structures (i.e., TENTs) which transverse the PC spaces of juxtapositioned BECs (see **yellow circles**). The TENTs are formed by cytoplasmic projections from the BEC membrane leading edges (Figure 1C). These cytoplasmic protrusions are continuous with the cell membrane material and extend across the intercellular cleft occluding the PC space (Figure 1D). For this study, we focused on the molecular cytoskeletal functionality of TENT development.

### 2.2. Cytoarchitecture of the BEC

**Figure 2:**
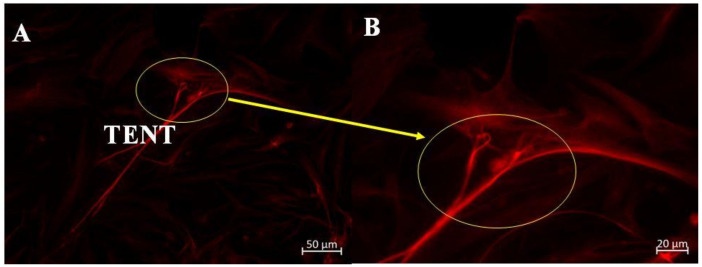
Alexa Fluor 568-stained α-tubulin of a non-treated bEnd3 cell. (**A**) α-tubulin interaction of cells, displaying the intramolecular profile of TENTs; (**B**) The magnification of the interaction of these α-tubulin-based TENT extending between the BECs membranes as indicated by the **yellow** circles.

In the scanning electron micrographs, we described the formation of TENTs between adjacent BECs (Figure 1). In the immunofluorescence (IF) study, we further investigated the role of Alexa Fluor 568-stained cytoskeletal α-tubulin as the intracellular backbone of TENTs. Figure 2A is a micrograph displaying the cytoplasmic projection of α-tubulin-rich slender microtubes between adjacent BECs. The α-tubulin within TENTs is able to project into the PC space, engaging the BEC membrane on the opposite end, and/or fully fusing with the opposite BEC membrane. Figure 2B, provides a higher magnification of the α-tubulin cytoskeletal elements of these foot-like distal ends of TENTs.

**Figure 3:**
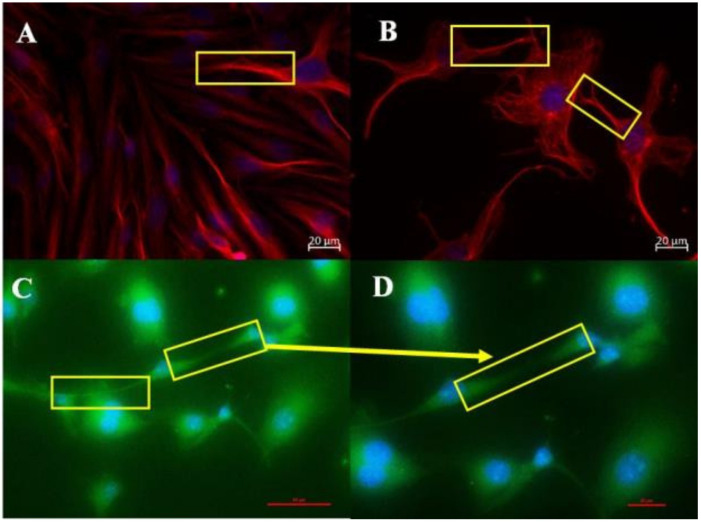
Immunofluorescence (IF) microscopy displays the α-tubulin and F-actin-rich cytoskeletal profile and molecular interaction between adjacent BECs that were grown on coated glass coverslips. (**A**) represents the intracellular molecular profile of α-tubulin-based cytoskeletal projections and the interaction between adjacent BECs; (**B**) Illustrates an extensive tubulin-based cytoarchitecture that is anchored inside the cell and α-tubulin-rich NT extensions from the leading edges of the BEC membranes; (**C**) Shows the intracellular molecular profile of F-actin-rich cytoskeletal projections between adjacent BECs; (**D**) Shows the magnification of F-actin-based NT extensions between adjacent BECs. The **yellow** squares indicate zones of α-tubulin interaction (**A**,**B**) and F-actin interaction (**C**,**D**) between adjacent BECs.

In both Figure 3A,B, α-tubulin is a cytoskeletal molecular structure that is found throughout the BEC. Figure 3A, shows the extensive α-tubulin-based scaffoldings inside the cell, with long, polymerized α-tubulin molecular structures forming the central cytoskeletal structure of the TENT. Figure 3B, demonstrates that TENTs utilize α-tubulin scaffolding within the soma of the BEC to anchor and project TENTs across the PC spaces of adjacent BECs. α-tubulin, therefore, appears to be a central component of TENTs anchoring substrate which allows tethering tubules to engage with the neighboring cells. TENTs, thus project out of the BEC with a molecular backbone of α-tubulin. In Figure 3C, F-actin is more extensively distributed across the BEC, forming extensive F-actin cytoskeletal scaffolding. We also clearly observe that F-actin that is anchored on the cytoskeletal scaffolding of the BEC extends into the TENTs, forming long polymerized F-actin filamentous structures. Figure 3D shows a magnified version of the propagating F-actin-based cytoskeletal extensions between the BECs that are growing in close proximity. 

### 2.3. Chemical Perturbation of the BEC TENT Morphology

#### 2.3.1. Effect of Cytochalasin D on TENT Morphology

**Figure 4:**
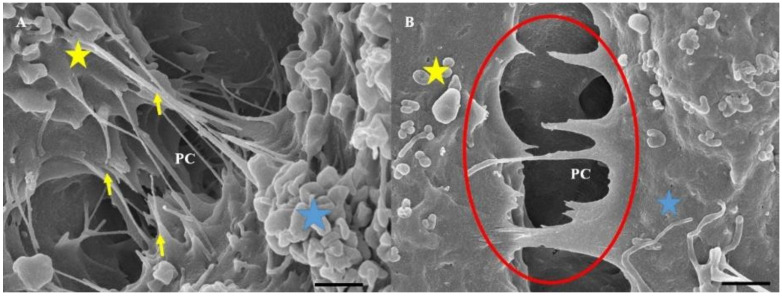
**High-resolution scanning electron microscopy** (HR-SEM) micrographical representation of the morphological deformation of cell-cell connections between adjacent BECs upon the chemical perturbation of actin polymerization utilizing a known depolymerizing agent, Cytochalasin D. (**A**) Indicates the numerous TENT interactions across the PC space between two bEnd5 cells. The micrograph clearly depicts the extensive formation of tethering-NTs which are naturally taut and project across the PC space; (**B**) A micrograph demonstrating the depolymerizing effect of Cytochalasin D on F-actin of bEnd5 cells where TENT formation is compromised forming thick, brittle-type NTs, as well as a distinct decrease in the TENT formation. An observable decrease in the number of NVs on the treated BEC membrane was seen, compared to the numerous NVs that were located on the control BEC membranes in (**A**). The **yellow** star represents cell 1, the **blue** star represents cell 2, and the paracellular space is denoted by **PC**.

The untreated BECs were able to generate intercellular TENT structures. The TENTs characteristically displayed focused development towards the targeted adjacent BEC, forming a cross-bridge scaffolding that results in a highly cross-linked PC space. In Figure 4B, treatment with Cytochalasin D resulted in an observable morphological distortion of intercellular TENTs and a decrease in BEC exosomal NVs. These exosomal NVs were prominently altered, in contrast to Figure 4A where the untreated BECs were able to generate numerous spherical NVs, which accumulated on the BEC membrane surface. 

**Figure 5:**
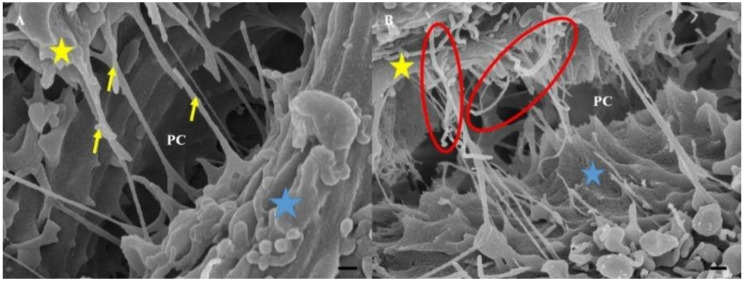
HR-SEM micrographical representation of the morphological deformation of cell-cell connections between adjacent BECs upon Nocodazole-induced cytoarchitectural depolymerization. (**A**) The bEnd5 TENT formation with cells that were grown in standard culture medium. The **yellow** arrows show the normal nature of PC TENTs, reflecting their bearing and tautness; (**B**) The effect of nocodazole exposure on bEnd5 NT formation relative to the control. The **yellow** star represents cell 1, the **blue** star represents cell 2, and the paracellular space is denoted by **PC**. The red circles in (**B**) indicate NTs which have lost their tautness and demonstrate the recoiling of TENTs in the Nocodazole-treated bEnd5 cell cultures.

The bEnd5 cells that were treated with Nocodazole failed to promote healthy NV formation and resulted in the formation of irregular shaped and flattened NVs that appeared to have collapsed on themselves. The number of cytoplasmic TENT projections in the Nocodazole-treated bEnd5 cells were distinctly less than the control (Figure 5) and displayed a PC space with a scanty NT network compared to the control conditions. 

#### 2.3.2. Effect of Nocodazole on TENT Morphology

In Figure 5A, the untreated bEnd5 cells displayed TENTs that formed tethered attachments between the plasma membrane of cell 1 to cell 2 across the PC space. Furthermore, the TENTs exhibited a characteristically taut appearance, forming a TENT scaffold between the BECs, facilitating cell membrane alignment. In Figure 5B, upon treatment with Nocodazole, the TENTs failed to form correctly. The TENT lost its ability to directly target the juxtaposed lateral plasma membrane across the PC space, failing to form a structured NT network between the BECs due to the recoiling of the TENT structures.

### 2.4. Effect of Chemical Perturbation on the BEC Cytoskeleton

#### 2.4.1. Effect of Cytochalasin D on BEC Cytoarchitecture

**Figure 6:**
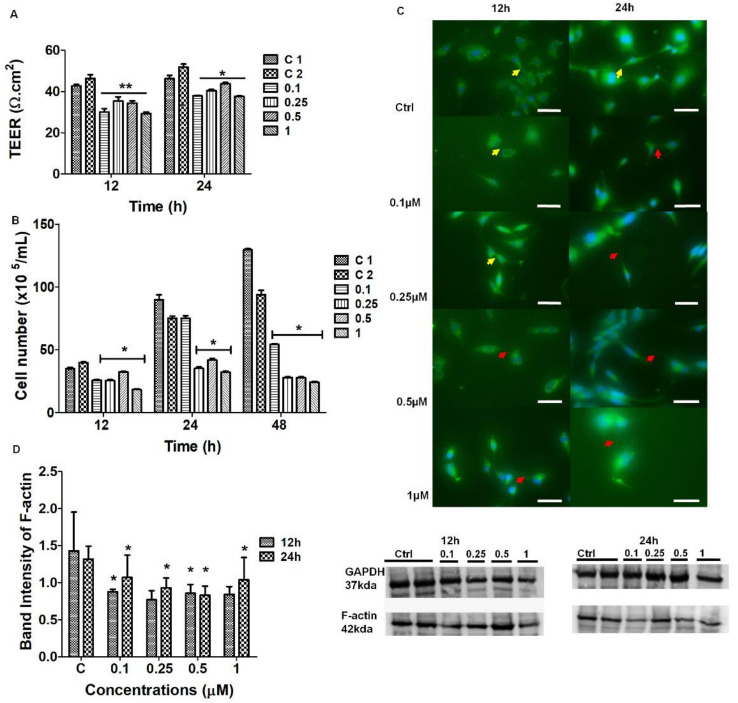
The effect of known depolymerizing agent Cytochalasin D on the physiological parameters of BECs. (**A**) The effect of 0.1–1 μM Cytochalasin D on the TEER across the confluent monolayers of bEnd5 BECs after 12–24 h exposure. The single asterisk * represents significant differences between the experiments and the control samples. The double asterisk (**) denotes statistical significance between the experimental samples and the vehicle control 2 (C2). The data are expressed as the mean ± SEM; (**B**) represents the effect of Cytochalasin D on bEnd5 cell numbers. The asterisks * denotes statistically significant differences between the experimental samples relative to control 1 (C1) and the vehicle control 2 (C2- with 0.1% DMSO); (**C**) Assessing the depolymerizing effect of Cytochalasin D in IF micrographs of Alexa Fluor 488 conjugated, monoclonal F-actin antibodies (Ab’), the **yellow** arrowheads indicate the regions of NT formation and the **red** arrowheads indicate the regions of depolymerization and/or intercellular gaps at the membrane leading edges between adjacent BECs, 12 h and 24 h. D (Scale bar = 50 μM) (**D**) Western blot analysis displays the effect of chemical perturbation of Cytochalasin D on F-actin expression. The asterisks * denotes statistically significant differences between the experimental samples and both control 1 and 2. The data are represented as the mean ± SEM (*n* = 3). Statistical significance was determined at *p* < 0.05.

To investigate whether F-actin played a role in maintaining the permeability status across the BEC monolayers, confluent monolayers of bEnd5 cells were treated with selected concentrations of Cytochalasin D. In Figure 6A, the transendothelial electrical resistance (TEER) studies across the BEC confluent monolayers showed an observable decrease in TEER across all concentrations of Cytochalasin D exposure compared to the untreated (control conditions) for 12–24 h. In Figure 6B, significant suppression in the cell numbers was observed (*p* < 0.001) from 12–24 h, relative to controls. At 24 h, the lowest concentration of 0.1 μM showed no significant effects, but upon increasing concentrations of Cytochalasin D, from 0.25 μM to 1 μM, a significant decrease in the cell numbers was observed relative to the controls (*p* < 0.01). In Figure 6C, IF micrographs display the observable progression in F-actin depolymerization between adjacent BECs. The F-actin cytoskeletal extensions became progressively diminished and direct cell-cell interaction between the BECs dissipated. In Figure 6D, quantitative Western blotting analysis showed that, at 12 h exposure to Cytochalasin D (0.1–1 µM) showed a decrease in the amount of the cytoskeletal, microfilamentous protein, F-actin, relative to the control samples. F-actin decreased in a dose-dependent manner, with significant differences observed at lower (0.25 μM) and higher (1 μM) concentrations (*p*-value < 0.0015). At 24 h, the lower concentrations (i.e., 0.1–0.25 μM) appeared to recover relative to the control samples, however, the overall expression of F-actin remained significantly depressed relative to the control samples (*p*-value < 0.0186). To investigate the effect of Cytochalasin D treatment on cell division in non-confluent bEnd5 cultures, the number of treated live cells were compared to the control cell cultures that were not treated, for 12, 24, and 48 h. 

#### 2.4.2. Effect of Nocodazole on BEC Cytoarchitecture

**Figure 7:**
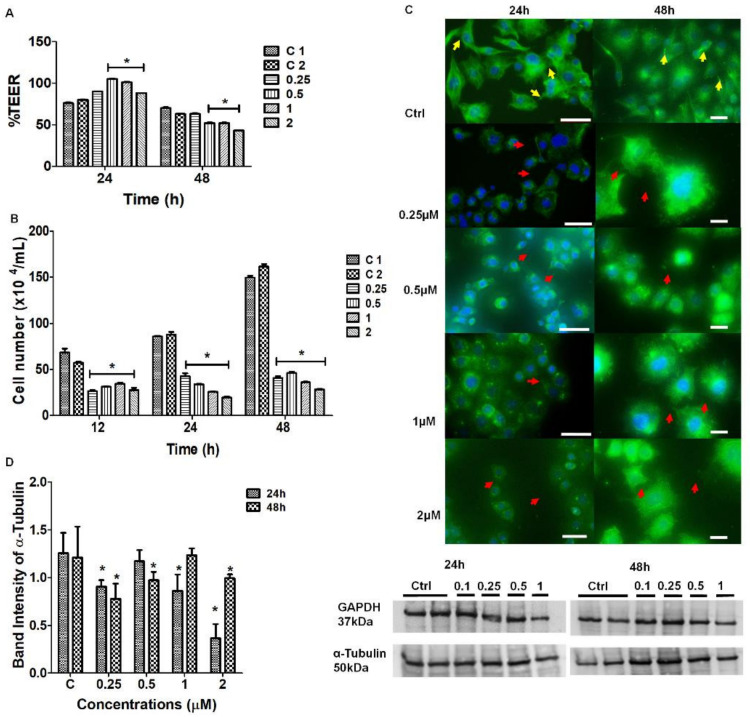
The effect of Nocodazole on the selected physiological parameters of bEnd5 cells. (**A**) The decreasing TEER across confluent bEnd5 cell monolayers upon treatment with 0.25–2 μM Nocodazole. The single asterisk * represents significant differences between the experiments and the untreated (control) samples. The data are expressed as the mean ± SEM; (**B**) Represents the effect of Nocodazole on bEnd5 cell numbers. The asterisk * denotes statistically significant differences between the experimental samples relative to the untreated conditions (C1) and the vehicle control 2 (C2-with 0.1% DMSO); (**C**) IF micrographs of Alexa Fluor 488 conjugated, monoclonal α-tubulin Ab’ after 24–48 h Nocodazole treatment for 24 h (Scale bar = 50 μM) and 48 h (Scale bar = 20 μM). The **yellow** arrowheads indicate regions of intercellular NT formation and the **red** arrowheads indicate regions of depolymerization at the membrane leading edges between adjacent BECs; (**D**) The depolymerizing effect of Nocodazole on α-tubulin at 24 h and 48 h observed in the Western blot analysis, which showed significant suppression in protein expression relative to the controls for 24–48 h. The asterisks * denotes statistically significant differences between the experimental samples and both control 1 and 2. The data are represented as the mean ± SEM (*n* = 3). Statistical significance was determined at *p*-value < 0.05.

To investigate whether α-tubulin played a role in maintaining the permeability status across the BEC monolayers, confluent monolayers of bEnd5 cells were treated with selected concentrations of Nocodazole. Figure 7A shows an increase in TEER across a confluent monolayer of bEnd5 BECs at 24 h, relative to the untreated samples. Conversely, a slight dose-related effect was evident at 48 h with a decrease in TEER upon increasing doses of Nocodazole. In Figure 7B, significant suppression in cell numbers was observed from 12–24 h across all concentrations of Nocodazole (0.25–2 μM) (*p* < 0.001), relative to the control samples. Figure 7C showed that the α-tubulin-rich population aligned along the leading edges of the BEC plasma membrane in untreated samples (controls), with effective cell-cell interaction (see the **yellow** arrows). Comparatively, the cells that were treated with Nocodazole did not exhibit α-tubulin alignment along the BEC membranous leading edges, but rather produced disorganized thread-like structures that were caused by depolymerization treatment. The depolymerization was more effective along the peripheral edges of the BEC plasma membrane (see the **red** arrows) (Figure 7C). The untreated samples displayed less pronounced polymerization at the cell center and an accumulation of α-tubulin around the nucleus. In Figure 7D, although the study was repeated in triplicate, there was no clear pattern of dose-related suppression in α-tubulin. The suppression of α-tubulin that was, however, observed relative to the control samples at 24–48 h (*p* < 0.0006), directly correlated with increasing concentrations of Nocodazole (*p* < 0.0016).

## 3. Discussion

BBB disruption is a characteristic feature of neurodegeneration [14] and alterations in BEC interactions are known to result in the failure of angiogenesis of brain capillaries with the ensuing disruption of the central nervous system (CNS) homeostasis. The metric for BBB integrity is largely founded upon its degree of impedance and selective permeability, but when it is compromised it no longer can prevent pathogens and harmful substances from crossing into the brain’s microenvironment. When investigating the permeability aptitude of the BBB interface it is critical to remember that it is still essentially a physical barrier that is primarily located at the level of the BEC. We, therefore, consider studying its physical status by exploring the HR-SEM micrographs the cellular morphology of confluent BECs at a nanoscopic level, together with the molecular cytoskeletal underpinning of these novel nanosized extracellular structures.

As more research has been implicating the BECs of the BBB as central to neurodegenerative processes [15], the importance of the BBB is that its regulatory function is closely related to the regulation of permeability through the occlusion of the PC space by TJ interaction. How these zones of juxtaposed transmembrane TJs align is crucial to their action. The transmembrane proteins are not arranged randomly across the lateral membranes of the adjacent BECs, but are arranged in narrow apico-lateral zones along the lateral borders of the BEC plasma membrane [16]. Varying morphology of adjacent BECs will result in misaligned TJ zones with juxtaposed TJs not being able to interact with each other, compromising the occlusion of the PC shunt. In view of the crucial nature of aligning zones of adjacent TJs, our HR-SEM micrographs (Figure 1) of the PC space present a view of a highly complex arrangement of NTs, which appear crucial to the formation of the PC space that would be sealed by TJs. These micrographs reiterate our previous work on the morphology of the BEC monolayers [2], by presenting the interplay of TENTs and TUNTs in the formation of a sealed PC space. In this study, we observed that cross-linking of the TENTs across the PC space seems crucial to the alignment of the lateral plasma membranes to facilitate the engagement of TJs. We wanted to further investigate what molecular cytoskeletal structures were at play in these TENTs and how these cytoskeletal structures were connected to the scaffolding cytoskeletal structures of the soma of the BEC. 

We know that transmembrane TJs are attached to cytoplasmic plaque proteins (zonula occludens-1,-2,-3) that are found embedded within the soma of the BEC cytoskeleton. These transmembrane proteins are interlinked to the cytoskeleton, which provides structural support for the TJ protein complex as well as orientation [1]. Understanding the underpinning molecular mechanisms that are governing the BEC cytoskeletal dynamics and subsequent morphology is central to the maintenance of cellular orientation, allowing for aligned brain capillary EC interaction and BBB establishment.

Although Mentor and Fisher [2] described the importance of NTs in this function and described their characteristics and morphology for the first time, the role of TENTs as a causative feature in barrier generation and impedance remains to be defined. In this study the authors postulate that TENTs have a direct role in the alignment of the PC spaces between adjacent BECs, enabling juxtaposed zones of TJs to align and interact, ultimately sealing off the PC spaces. The authors proceeded to unpack this postulate, by asking “what cytoskeletal proteins are central to TENT formation and do they play a physiological role in BBB function?”

Amidst the myriad of intercellular communication, namely (i) paracrine communication; (ii) direct cell-cell contact, mediated in some cell types by gap junctions and by synaptic communication in neurons [17,18]; and (iii) direct cellular–cell communication, by way of cytoplasmic projected filipodia [9,19,20], a novel form of direct BEC–BEC communication via TUNT and TENT formation that is based on a recent HR-SEM study by Mentor and Fisher [2] has emerged. For this study, however, we have undertaken IF imaging and selected the perturbation of the polymerization of F-actin and α-tubulin approach, to understand the functional imperative of the TENTs in the developing *in vitro* BBB model [2].

### 3.1. Morphological Studies

#### 3.1.1. Effect of Cytochalasin D on TENT Morphology

Cytochalasin D is known to block the process of F-actin polymerization by binding to the barbed (+) ends of the actin microfilaments, inhibiting its elongation [21]. This preventative action stunts cell cytokinesis (i.e., cell division following mitosis), thus actin is critical for these processes. An observable decrease in TENTs was observed on HR-SEM micrographs after treatment with Cytochalsin D. The depolymerizing effect of Cytochalasin D may have resulted in the inhibition of TENT formation across the PC space, preventing its leading edges from extending into intercellular NT cross-bridges between adjacent BECs (Figure 4A,B).

#### 3.1.2. Effect of Nocodazole on TENT Morphology

The treatment of bEnd5 cells with Nocodazole is a known inhibitor of α-tubulin polymerization, resulting in a loss of tautness and/or distortion in TENT formation between adjacent BECs, compared to untreated samples (controls) (Figure 5). This observation was further supported by observations of the backward folding of the TENTs, in the opposite direction of the neighboring cells (Figure 5B) relative to untreated samples (Figure 5A). The findings support the postulate that the distortion of TENTs is directly affected by the exposure to the depolymerization agent which affected its stability resulting in the failure of its directional ability to extend across the PC space and fuse with the plasma membrane of an adjacent cell. The prevention of functional NT tether formation results in the failure to mechanically engineer a convoluted/interactive PC space, ultimately preventing direct cell–cell engagement between adjacent BECs growing in close proximity during BEC bEnd5 monolayer establishment. 

### 3.2. Cytoskeletal Studies

We used IF to investigate the cytoskeletal molecular structures in the BEC and its projection into TENTs. The IF micrographs show that the soma of the BEC has an extensive network of F-actin and α-tubulin. In the control samples, F-actin is interconnected throughout the cell cytoplasm, forming cylindrical bundles of long spiral chains while α-tubulin-based microtubules pervade the cytoplasm of the BEC, forming long chains; both form the vital components of BEC intracellular cytoarchitecture (Figure 2A,B). Furthermore, it is well known that F-actin-based microfilaments are important for actuating the endothelial barrier function and α-tubulin-rich microtubules are ubiquitous within the cell’s cytoskeleton and protrude from the cells surface [8], alluding to its ability to influence the underlying mechanism of the BEC vascular barrier establishment. Our studies support this view in that the cytoskeletal molecular composition of BEC cytoplasmic extensions, TENTs, was shown to possess both F-actin and α-tubulin-rich cytoskeleton (Figure 3A,B). The microfilaments are actin-rich and are associated with myosin proteins which promote cellular movement, contraction, and cytokinesis, whereas the microtubules, which are tubulin-rich, are tubular structures that are involved in promoting cell shape, transport, and activation of the cells actin-cytoskeleton via guanosine triphosphatases (GTPases) (i.e., Rho and Rac) [12,22].

#### 3.2.1. Effect of Cytochalasin D on the Cytoskeletal Architecture of the TENT

The chemical perturbation of cytoskeletal proteins involved treating BECs with Cytochalasin D (0.1–1 μM) to depolymerize F-actin (Figure 6). The IF micrographs exhibited a reduction in F-actin protein expression which resulted in the failure of BECs to maintain direct cell-cell contact via cytoplasmic TENT extensions (Figure 6C). These findings were endorsed by Western blot analysis which confirmed that Cytochalasin D was able to cause a dose-related suppression in the F-actin protein due to the reduction in the protein content of F-actin within the BEC cytoplasm (Figure 6D).

It is well-known that the actin-cytoskeleton plays a functional role in cell-cell contact [23]. Cytochalasin D is well known to cause the depolymerization of the F-actin-based microfilaments. The F-actin cytoskeletal protein interlinks TJ scaffolding in the cytoplasm of cells by interacting with the ZO-1 plaque proteins within the BEC [24]. Our findings suggest that any alterations in F-actin will directly affect the transmembrane TJ protein arrangement, but also compromise TENT dynamics. Our morphology study endorsed this view in that the chemically-induced alteration of the TENTs resulted in the failure of adjacent BECs to align, orientate, and juxtapose with each other to initiate apico-lateral TJ interaction. Furthermore, the IF micrographs showed a decrease in the cytoskeletal F-actin expression, consequently affecting the degree of direct BEC–cell interaction by way of cytoplasmic F-actin protrusions on the BEC peripheral borders/leading edges of the BEC, further supporting this view. The role of F-actin appears to not only be crucial to the BEC soma morphology, but also to TENT structure and function. The depolymerization, reduction of cross-linking, disorganization, and defragmentation of F-actin-rich microfilaments could result in failed TJ anastomosis and consequently compromise the impermeability of the BECs PC spaces. 

#### 3.2.2. Effect of Nocodazole on the Cytoskeletal Architecture of the TENT

The findings in the IF studies of cytoskeletal protein α-tubulin revealed that Nocodazole, at a concentration range of 0.25–2 μM, caused both a reduction and disorganization in the arrangement of α-tubulin from its native state (Figure 7C). The distortion of the long, slender, α-tubulin-rich microtubules resulted in the failure of BEC to form membranous protrusions and subsequent cell-cell adhesion between adjacent cells that were growing in close proximity. In Figure 7D, Western Blot data and analysis showed no definite pattern and/or clear effect. Furthermore, there was no relationship between the permeability (TEER data) (Figure 7A) and protein expression. Tubulin heterodimers are the monomers of the intracellular microtubules, which are ubiquitous within the cytoskeletal backbone of all cells [25]. It is important to note that the tubulin-rich microtubules are functionally related to transport, motility, and cytokinesis during cell division [26,27]. Any structural alterations in this protein are directly associated with subcellular cytoskeletal rearrangement which directly impacts the cell morphology, disrupting the optimal functionality involved in achieving cell-cell contact which is critical for a strictly regulated barrier [23,26,28] 

HR-SEM and IF studies showed that Nocodazole caused a radial reduction in α-tubulin expression (Figure 7C) directly implicates the BECs ability to make direct cell–cell contact, further contributing to the misalignment of BEC lateral plasma membranes, leading to the failure of TJ juxtaposed zones to align. The *in vivo* BBB requires the interaction of actin microfilaments with plaque proteins zonula occludens-1, -2, -3 (ZO-1, -2, -3) to ensure the formation of a polarized brain endothelium which ultimately drives BBB development [26]. Thus, a compromised barrier functionality of BECs implicates brain microvascular pathology and is closely linked to cytoskeletal rearrangements and actomyosin contractility, subsequently resulting in the formation of increased PC permeability between BECs. 

### 3.3. Permeability

Barrier-genesis is central to this study. This study evaluates how F-actin and α-tubulin play a major role in NT structure and directly implicates TENTs in the regulation of permeability across the *in vitro* BBB model.

#### 3.3.1. The Effect of Cytochalasin D on BEC Permeability

The TEER studies focus on the interaction of BECs with the context of a confluent monolayer, and the effects on permeability (TEER) after treatment with a depolymerizing agent. At 12–24 h, the BEC bEnd5 monolayers showed a significant, dose-dependent increase in BBB permeability (*p* < 0.01) for all concentrations of Cytochalasin D, relative to the untreated samples (*p* < 0.001) (Figure 6A). Based on these findings, we can infer that blocking the depolymerization of F-actin affects TENT formation directly by implicating the physiological prowess of BEC interaction and subsequent barrier-genesis as seen by increased permeability across our *in vitro* BBB model.

#### 3.3.2. The Effect of Nocodazole D on BEC Permeability

Upon treatment with Nocodazole, the BEC TEER studies showed that within the first 24 h, the permeability increased relative to the untreated samples. At 48 h the experimental samples exhibited a significant decrease in permeability (*p* < 0.001) at the higher concentrations of Nocodazole, relative to the untreated samples (Figure 7A). It is well reported that increased vascular permeability is associated with increased pathology, especially after the treatment with anti-cancer mitosis-blocking therapy [23]. Smurova et al. [23] reported on the ability of 100–200 nM Nocodazole to disrupt microtubules at the cell margins and transiently increase the EC permeability at the lower concentrations.

The increases in TEER within the first 24 h can be correlated with the expression of F-actin at 24 h within the IF studies. However, at 48 h there was a reduction in protein expression at the highest dose of Nocodazole, which did not parallel the decrease in TEER at the same concentration. These findings are further supported by a study conducted by Eshun-Wilson et al. [25] who reported that the state at which α-tubulin depolymerization occurs depends largely on whether the protein of interest has undergone post-translational modification through the acetylation of lysine 40 loop (K40) in α-tubulin. These conformational changes are reported to improve microtubule stability, reducing the disorder of the loops [29,30,31]. The variability in the rate of depolymerization of α-tubulin in TENTs that were observed in our study may, therefore, be dependent on whether the protein has undergone post-translational acetylation. Our α-tubulin data may be more varied upon treatment with Nocodazole due to the effects of post-translational acetylation.

It is conspicuous in contrasting the effects of Cytochalasin D and Nocodazole on the permeability across confluent bEnd5 monolayers, that depolymerization of F-actin had a pronounced effect of increasing the permeability, whereas depolymerization of α-tubulin had a varied effect on permeability. This suggests that F-actin and α-tubulin affect permeability across the BEC monolayers by different mechanisms. In tubular cellular structures, α-tubulin plays an important role in intracellular transport viz, in axons via the plus end-directed kinesins for anterograde transport, and dynein in dendrites [28,29,30]. It is, therefore, not difficult to postulate that the role that α-tubulin plays in TENTs may be related to the transfer of molecular signaling between adjacent BECs. This is endorsed by morphological evidence that indicates that the distal ends of TENTs fuse with the lateral membrane of the adjacent BEC, essentially linking the cytoplasm of the adjacent BECs. F-actin on the other hand, plays an important role in the cytoskeletal structure of BECs and is intimately involved in anchoring TJ proteins to the intracellular actin-based scaffolding via the linking proteins ZO-1/2/3 [32]. We, therefore, tentatively postulate that F-actin that is found in TENTs are more involved with the structural rigidity of the NT structure and plays an important role in both stabilizing the PC space mechanically and aligning adjacent BECs to facilitate TJ interaction. The actin-based microfilaments are localized beneath the cells’ membrane, providing support to the cell’s shape, promoting the movement of the cells surface (cytokinesis), migration, and cell division [33]. Thus, the ability of the BBB to form a well-regulated barrier will be compromised if the structural orientation of the BEC is altered, resulting in the potential progression of cerebromicrovascular pathology. 

### 3.4. Effects of Cytochalasin D and Nocodazole on BEC Cell Division

This study investigated how selected inhibitors of polymerization affect BEC division. In this study, in contrast to confluent monolayers that were used in the morphology and TEER studies, non-confluent cell cultures were evaluated to elucidate the effect of depolymerizing agents on cell division. We monitored cell division of BECs, which is essential for the normal maintenance and regeneration of the BBB (e.g., especially after cerebrovascular accidents, infections, and/or inflammation), after treatment with Cytochalasin D and Nocodazole. Cytochalasin D affected BEC proliferation by causing a suppression in BEC division, relative to the untreated samples (Figure 6B). Similarly, Nocodazole treatment resulted in a significant suppression in BEC division (Figure 7B). All the treated cells showed significant suppression in BEC proliferation, thus, inferences can be made that the inhibitory effect of the compound on BEC numbers could be directly attributed to the ability of Nocodazole to disrupt α-tubulin polymer formation in a manner that negatively impacts the rate of BEC division (Figure 7B). These findings are endorsed in the literature, which reports that Nocodazole has the ability to inhibit mitosis and it is well reported that a critical requisite for cell division is appropriate mitotic spindles during mitosis [34]. The inhibition of microtubules is reported to result in cell-cycle arrest at the G_2_-M phase, with the formation of abnormal mitotic stimulants [34,35] and thus, our results endorse these findings in that Nocodazole resulted in an overall suppression in BEC division. 

### 3.5. Synthesis

This study is focused on the concept of barrier-genesis of the brain capillary endothelium and is specifically focused on the role and mechanisms whereby TENTs affect barrier establishment of the *in vitro* BBB model. The findings showed the integral role of TENTs in BEC monolayer formation and, thus, by extrapolation, must form an integral part of an *in vivo* BBB. By logical extension, BECs that are grown close together form a strict monolayer, which is a reflection of the *in vivo* endothelium of the brain capillary. This *in vitro* complexity cannot, therefore, be an aberration of the *in vivo* scenario, but is rather a reflection of the complexity within the *in vivo* state. We found that the polymerization of F-actin and α-tubulin protein structures were ubiquitous within the TENTs. Upon chemical perturbation with Cytochalasin D and Nocodazole, we saw a breakdown in these protein structures using IF, which was supported by the morphological loss of tautness and an increase in the morphological distortion in the TENT structures. To examine how depolymerization on barrier functionality we measured TEER across the BEC bEnd5 confluent monolayers as an index for measuring the barrier integrity. TEER was affected by F-actin breakdown, whereas tubulin breakdown generated variable TEER results. Based on these findings we can infer that F-actin in TENTs is more essential for barrier-genesis than α-tubulin. Conversely, as the effects of α-tubulin on TEER were inconclusive, we propose that α-tubulin in TENTs play a role in conducting molecular signaling between adjacent BECs, as is evident by its role in other cell structures such as in axonal transport in neurons [29]. 

Despite TENT structures constituting both microfilaments F-actin and α-tubulin, the role of these cytoarchitectural proteins differ in terms of their direct/indirect involvement in barrier-genesis. The physical functionality of a TENT involves extensive cytoskeletal governance to ensure that adjacent BEC plasma membranes are aligned into position, allowing for TJ localization and the occlusion of the PC spaces during BBB establishment. 

## 4. Materials and Methods

### 4.1. Cell Culture

The bEnd5 cell line was purchased from the European Collection of Authenticated Cell Cultures (ECACC) (Sigma-Aldrich, 96091930, St. Louis, MI, USA). The cells were cultured in Dulbecco’s Modified Eagles medium (Whitehead Scientific, Cat no. BE12-719F, Stikland, South Africa), supplemented with 10% fetal bovine serum (FBS) (Celtic Molecular Diagnostics /Biowest, Cat no. S181G-500, Cape Town, South Africa, 1% Penicillin/Streptomycin (Whitehead Scientific, Cat. No. DE17-602E, South Africa), 1% non-essential amino acids (Whitehead Scientific, Cat no. BE13-114E, South Africa), and 1% sodium pyruvate (Whitehead Scientific, Cat no. BE13-115E, South Africa).

### 4.2. Transendothelial Electrical Resistance

A total of 24 h after seeding, 5 × 10^5^ bEnd5 cells/insert/well on membrane inserts (Millipore/Merck, Cat no. PIHA01250, Darmstadt, Germany), in 24-well plates until they reached confluence and the cultured medium was replaced by select concentrations of depolymerizing agents: Cytochalasin D at 0.1 μM, 0.25 μM, 0.5 μM and 1 μM, relative to control samples. Nocodazole at 0.25 μM, 0.5 μM, 1 μM, and 2 μM at the humidified atmosphere of 5% CO_2_ at 37 °C. Transendothelial electrical resistance (TEER) was carried out for 12 h, 24 h, and 48 h using a Millicell electrical resistance system (Millipore, Ser. No. 57318 11B, Germany). Quantitative analysis of the recorded TEER readings was achieved by employing the parameters of an appropriate equivalent circuit, which represents the electrical parameters across the *in vitro* BEC monolayer under investigation. The TEER values are expressed in Ω·cm^2^ and were normalized to controls and plotted as percentage normalized resistance.

### 4.3. Scanning Electron Microscopy

A total of 48 h after seeding and exposing, the bEnd5 cell monolayers cells on inserts were fixed with 2.5% glutaraldehyde (Fluka/Sigma, Cat no. 49626, Darmstadt, Germany) prepared in 1X phosphate-buffered saline (PBS) (Life Technologies, Cat. no. 20012019, South Africa) for 1 h at room temperature (RT). After a thorough rinse with 1X PBS and water, the cells were dehydrated in ethanol (50–100%). This was followed by a critical point drying step for 1 h, in which ethanol was replaced by CO_2_, and finally sputter coated with gold:palladium (Au:Pd). The samples were visualized by a scanning electron microscope (Zeiss-Auriga, Erfurt, Germany).

### 4.4. Immunocytochemistry

Immunocytochemistry detects antigens (Ag) in tissue sections utilizing immunological and chemical reactions. It is highly sensitive and specific and is able to detect a variety of protein-specific Ag in multiple animal species. The bEnd5 cells were seeded at a density of 50,000/well, on gelatin-coated glass coverslips in 12-well plates. After 24 h following cellular attachment, the cells were treated with depolymerizing agents: (i) Cytochalasin D at 0.1–1 μM and (ii) Nocodazole at (0.25–2 μM). After exposure to the drug at the respective time intervals, the samples were fixed with 4% paraformaldehyde (made in PBS pH 7.4) for 10 min at RT. The BECs were washed thrice with ice-cold 1X PBS for 5 min each. The BECs were incubated in 1% bovine serum albumin (BSA) (Sigma, Cat no. 05470-1G, Germany) which was prepared with 0.1% Triton X (Sigma-Aldrich, cat no. 9002-93-1, Germany) in PBS) for 1–2 h to permeabilize the cell membrane. Thereafter, the cells were incubated in 1%BSA, the purpose was to block unspecific binding of the antibodies. A 3% BSA solution (blocking solution) was prepared by dissolving 1.5 g BSA into the 50 mL PBS solution with 0.1% Triton X. The 0.1% Triton X solution was prepared by dissolving 50 μL in 50 mL of 3% BSA.

### 4.5. Immunostaining

After a 1–2 h time-lapse, the blocking solution was removed and the primary antibody (Ab`) for F-actin (1:1000) (Life Technologies/ThermoFisher Scientific, Cat no. MA180729, City of Johannesburg, South Africa) and α-tubulin (1:200) (Merck Chemicals, Cat no. T8203, South Africa) was added to semi-confluent bEnd5 monolayers that were grown within 12-well plates. The Ab` was incubated on a shaker for 1h at room temperature (RT) or overnight at 4 °C. The Ab`s were removed and the cell samples were washed with PBS for 5 min (min) and repeated three times. Thereafter, the cell samples were incubated with the following secondary Ab`: Alexa Fluor 488-conjugated goat anti-mouse secondary Ab` (1:500) (Life Technologies/ThermoFisher Scientific, Cat no. A-11001, South Africa) and Alexa Fluor 568-conjugated goat anti-mouse secondary Ab` (1:500) (Life Technologies/ThermoFisher Scientific, Cat no. A-11011, South Africa) for 2 h. Thereafter, the secondary Ab` solution was removed and the cells were washed for 5 min with PBS three times (this step was performed under dark conditions to avoid bleaching of the fluorescent Ab`).

### 4.6. Counterstaining with 4’,6-Diamidino-2-Phenylinole (DAPI) Stain

The DAPI stain is a blue-fluorescent stain targeting double-stranded deoxyribonucleic acid (dsDNA). The binding of DAPI to dsDNA produces a ~20-fold fluorescent enhancement; this is attributed to the displacement of water molecules from both DAPI and the minor groove of the dsDNA (Larson et al., 2012). DAPI remains a popular fluorescent stain that is employed for DNA visualization and quantification. The cells were incubated with 0.1–1 μg/mL DAPI (fixed cells, grown on glass slides). A total of 1 µL of DAPI (ThermoFisher Scientific, Cat no. 62248, South Africa) was added to 10 mL of 1X PBS (Store at 4 °C under dark conditions). The DAPI stain was exposed to the fixed cells under dark conditions for 10 minutes and, thereafter, washed in 1X PBS (three times, for five minutes each). 1,4-Diazabicyclo[2.2.2]octane (DABCO) fluorogel (Sigma-Aldrich, Cat no. D27802-100G, Germany) was added to a glass slide for mounting the cell samples that were grown on a glass coverslip. The samples were viewed using immunofluorescent (IF) microscopy (Nikon Eclipse Ts2).

### 4.7. Western Blot Analysis

The bEnd5 cells were allowed to grow in 75 cm^3^ tissue culture flasks and were treated with different concentrations of Nocodazole (0.25 μM, 0.5 μM, 1 μM, and 2 μM) and Cytochalasin D (0.1 µM, 0.25 μM, 0.5 μM, and 1 μM) for 12 h, 24 h, and 48 h. The cells were washed in 1X PBS and trypsinized in 0.25% trypsin versene–EDTA (Whitehead Scientific, Cat no BE17-161E, South Africa). The samples were pipetted in microcentrifuge tubes and centrifuged at 1500 revolutions per minute (rpm) for 5 min RT and the supernatant was discarded. The pellet was resuspended in cold 1X PBS, on ice and centrifuged at 4000 rpm for 5 min at 4 °C. A total of 140 µL of lysis buffer was prepared by adding 250 µL of phosphatase inhibitor cocktail and 250 µL protease inhibitor cocktail to 5 mL of RIPA buffer (ThermoFisher Scientific, Lot no. VI311029 was added to each cell pellet on ice and lysed by agitation for 10–15 min, thereafter, the lysate was sonicated and centrifuged at 14,000 rpm for 10 min. The protein concentration was determined using the Thermo Scientific Nanodrop 2000/2000c spectrophotometer (ThermoFisher Scientific, USA). 

The sample solutions were diluted using Laemli sample buffer (LSB) (Biorad, Cat no. 1610737) and 5% Beta mercaptoethanol (Biorad, Cat no. 1610710) to normalize the protein concentrations to 20µg/mL and denatured at 95 °C, for 5 min. The samples were run on the 10% SDS-PAGE gel at 200 V (400 mA) for about 45–50 min or until the bottom-most marker band reached the bottom of the gel. The proteins were transferred to nitrocellulose membranes for 7 min at 20 V at RT using the iblot2 transfer system (ThermoFisher Scientific, Ref. no. IB23001, South Africa) and the membranes were blocked for 2 h using 2% bovine serum albumin (BSA) or Casein blocking solution. The membranes were probed with primary monoclonal ab F-actin (1:1000, Thermofisher Scientific, USA), α-tubulin (1:2000, Sigma, Darmstadt, Germany) and GAPDH (1:4000, Invitrogen, Burlington, ON, Canada) and incubated overnight. The membrane was incubated with secondary antibody horseradish peroxidase (HRP)-conjugated goat anti-mouse secondary ab (1:10,000) for 2 h and washed three times with PBS-tween. The membrane was subjected to substrate chemiluminescence for 5 to 15 min and the BioRad ChemiDoc imaging system, version 2.4.0.03 (Lasec, South Africa) was used to view the band formation. 

### 4.8. Trypan Blue Exclusion Assay

A cell density of 5 × 10^4^ bEnd5 cells/well in 24-well plates were seeded and allowed to reach confluence over a 24h timeframe. Thereafter, the culture medium was replaced by the selected concentrations of depolymerizing agents: Cytochalasin D at 0.1 μM, 0.25 μM, 0.5 μM, and 1 μM, relative to the untreated (control) samples at 12–24 h; and Nocodazole at 0.25 μM, 0.5 μM, 1 μM, and 2 μM, relative to the control samples for 24–48 h, at the humidified atmosphere of 5% CO_2_ at 37 °C. The cellular proliferation was investigated by utilizing the trypan blue exclusion assay to determine the effect of the selected depolymerizing agents on the rate of BEC division. A cell count was performed using the Countess III automated counter (Invitrogen), which employs built-in standardized algorithms that allow for the elimination of debris, considers cell cluster formation, and accounts for the cell size.

## 5. Conclusions

This study addressed the functional role that TENTs play in establishing a strictly regulated BEC monolayer. In view of the effect of F-actin treatment on confluent monolayers which produced an increase in the permeability (decrease TEER), we postulate that F-actin provides cyto-structural focus and tautness to the TENT structure. In contrast, the α-tubulin in TENTs, in addition to its structural function, as could be seen in our morphology study, does not play as prominent a role in permeability (TEER) integrity. We, therefore, postulate that it may play a role in direct cell-cell signaling across the PC space. Thus, based on the ultrastructural and cytoarchitectural findings we postulate that TENTs not only provide tethering alignment of the PC space and TJ zones to occlude BEC PC spaces, but also play a role in direct cell-cell communication during BEC bEnd5 monolayer development. Moreover, compromising TENT formation by chemically perturbing the microfilaments and microtubules displays the dual importance of cytoskeleton proteins in providing both cell shape and transitions within BEC leading edges to form cell-cell contact points during barrier-genesis. The depolymerization of F-actin and α-tubulin negatively altered the intracellular cytoarchitecture of the BEC, changing its morphology and ability to form a well-structured BEC monolayer. The cytoskeleton is a salient feature of TENT formation as the cytoskeleton extends from the BEC soma into the TENT cytoplasmic extensions. Compromising TENT formation leads to the misalignment of adjacent BEC TJ anastomosis resulting in the failure to establish a well-regulated brain-endothelial barrier. This alludes to the integral role of NTs in monolayer development and, by extension, must form an integral part of the establishment of the *in vivo* BBB model. The morphological findings for TENT structures endorses the view that our *in vitro* BBB model is a promising method for studying BBB development. Our research, therefore, addresses a fundamental gap in how these novel NTs, TENTs play a role in the generation of the BBB endothelium in brain capillaries.

Furthermore, many neurodegenerative diseases are known to implicate BBB permeability, disrupting CNS homoeostasis. From a future perspective, we show the functional features of TENTs in BBB permeability and the usefulness of this *in vitro* BBB model may be important to elucidate the importance of TENTs in neurodegenerative disease progression.

## Data Availability

The data is archived according to UWC policies. The data presented in this study are available on request from the corresponding author.
